# Effects of Intense Pulsed Light Treatment on Dry Eye Disease After Keratorefractive Surgery: A Retrospective Cohort Study

**DOI:** 10.7759/cureus.86562

**Published:** 2025-06-22

**Authors:** Chia-Yi Lee, Shun-Fa Yang, Yun-Chen Chen, Chao Kai Chang

**Affiliations:** 1 Ophthalmology, Institute of Medicine, Chung Shan Medical University, Taichung, TWN; 2 Oncology, Institute of Medicine, Chung Shan Medical University, Taichung, TWN; 3 Ophthalmology, Chaomuhean Clinic, Taipei, TWN; 4 Ophthalmology, Taipei Branch, Nobel Eye Institute, Taipei, USA

**Keywords:** dry eye disease, intense pulse light, keratorefractive surgery, ocular surface, tear break-up time

## Abstract

Purpose: This study aimed to evaluate the therapeutic outcome of intense pulsed light (IPL) treatment in individuals with dry eye disease (DED) who received previous keratorefractive surgery (KRS).

Methods: A retrospective cohort study was conducted, and individuals who received IPL treatment were enrolled. Then, the individuals were categorized according to whether they underwent KRS, with a total of 11 and 35 eyes enrolled in the KRS and non-KRS groups, respectively. The primary outcomes were non-invasive tear break-up time (NITBUT), the Schirmer I test, ocular surface staining, and DED symptoms. A generalized linear model was used to evaluate the difference in treatment outcomes between the two groups.

Results: Six months after the IPL treatment, the post-treatment NITBUT was significantly higher in the non-KRS group than the KRS group (P < 0.001). Regarding the trend of DED improvement, NITBUT (P < 0.001) and ocular surface staining (P = 0.001) presented a better improvement in the non-KRS group. In regard to pre-treatment risk factors for poor response after IPL treatment, low NITBUT, low TMH, high ocular surface staining, and multiple DED-related symptoms correlated to poor treatment outcome in the KRS population (all P < 0.05). On the other hand, low NITBUT and high ocular surface staining were associated with poor treatment outcome in the non-KRS population (both P < 0.05).

Conclusions: Individuals with DED that previously underwent KRS presented a lower NITBUT and ocular surface staining recovery after IPL treatment compared to the non-KRS DED individuals, and KRS individuals have more risk factors for poor outcomes after IPL. Alternative DED treatment may be considered for the population with previous KRS.

## Introduction

Dry eye disease (DED) is a prevalent disease that affects many people and can be divided into the evaporative-excess, mucin deficiency, aqueous deficiency, and mixed types [1]. The subtype of DED would affect the treatment choice of the physician. For more details, meibomian gland management is needed for the evaporative excess-type DED, and the artificial tear supplement is warranted for the aqueous deficiency-type DED [1]. In the aspect of epidemiology, the prevalence of DED in the Asian region was slightly above 20% [2]. Common DED-related symptoms include dryness, a discharge sensation, grittiness, a foreign body sensation, a burning sensation, and photophobia [3]. Severe DED may contribute to reduced vision and impaired visual acuity [4], and keratitis could occur in individuals with advanced DED due to the damage to the ocular surface and the high level of inflammatory cytokines [5].

In regard to the current approach to DED, several medical and surgical treatments have been used [6]. Artificial tears have been frequently used for simple DED and postoperative DED in previous research [7]. Hyaluronic acid-containing artificial tears have become popular due to their anti-inflammatory function and high viscosity, which can significantly reduce DED signs and symptoms [8, 9]. Moreover, steroid eyedrops have been used to treat DED with good effects, and the concurrent administration of artificial tears and topical steroids will significantly improve DED symptoms with adequate safety [10]. In chronic DED, surgery such as amniotic membrane transplantation could be used to improve DED symptoms [11].

Intense pulsed light (IPL) treatment has been used to relieve DED for about 20 years [12]. The thermal effectiveness of IPL treatment is adequate for DED symptom reduction, especially in patients with evaporative-excess DED [13, 14]. In addition, IPL use showed a moderate post-keratorefractive surgery (KRS) DED management ability in previous publications [15, 16]. Still, there was scant research that investigated the effectiveness of IPL treatment between DED patients who underwent KRS and those with general DED that did not. In addition, the pathophysiology of KRS-related DED is different from general DED [17]. The effectiveness of IPL treatment in such a population may be different, and we hypothesize that IPL will be less effective for post-KRS patients.

Consequently, this study aims to compare IPL treatment results in general DED patients versus those post-KRS. The risk factors for poor responses after IPL treatment between the two groups were also analyzed in this study.

## Materials and methods

Participant selection

A retrospective cohort study was carried out at the Nobel Eye Institute, which is a group that operates more than 10 clinics in Taiwan. Individuals were selected if they met the following criteria: (1) were aged 20 to 100 years old, (2) were diagnosed with DED at the Nobel Eye Institute according to low tear break-up time (less than 10 seconds, which is an official standard for severe/prominent DED) and had subjective symptoms (i.e., dryness, grittiness, a foreign body sensation, and tearing, which are commonly found in DED patients in clinical practice), (3) underwent the automatic ocular surface analyzer test at the Nobel Eye Institute, (4) had IPL treatment arranged for them at least three times at the Nobel Eye Institute, and (5) visited the Nobel Eye Institute after IPL treatment for at least six months. In addition, the following exclusion criteria were used to eliminate individuals with debilitated ocular surfaces, which may complicate the assessment of DED condition and IPL effectiveness: (1) pre-existing central corneal opacity, (2) pre-existing corneal ulcer, (3) pre-existing central corneal erosion, (4) pre-existing deep corneal infiltration, (5) pre-existing corneal perforation, (6) pre-existing chemical burn that involved the central cornea, (7) pre-existing cicatricial conjunctivitis, and (8) the existence of systemic inflammatory disease, including, but not limited to, Sjögren's syndrome, rheumatic arthritis, systemic lupus erythematosus, diabetes, and thyroid eye disease. Then, the study population was further divided into the KRS group and the non-KRS group, which depended on whether the patients had previously undergone KRS. All the KRS procedures were laser in situ keratomileusis surgery, and all the patients received the surgery at least one year before the assessment. The right eye of each DED individual was assessed in this study. After the selection, a total of 11 and 35 eyes were included in the KRS group and the non-KRS group, respectively.

IPL therapy

All the IPL treatments were completed by one DED specialist (Y.-C.C.) using one device (M22TM, Lumenis Be Ltd., Yokneam Industrial Park, Yokneam, Israel), and three sessions were arranged with a total period of three months for all the patients. A local anesthesia gel was applied to the upper nasal area, upper eyelid area, and lower eyelid area, and the individuals waited for three minutes. A protective shield was then applied to the cornea before beginning the IPL treatment. Next, an 8x15mm square IPL probe was used on the right upper eyelid region first, and the IPL pulsed 10 times with an energy of 15J/cm², and the same energy was utilized on the right lower eyelid area for another 10 pulses. After finishing with the right eyelid, the left eyelid underwent the same procedure, and IPL treatment for both eyelids was performed again. After the IPL treatment, the individual was taken to a slit-lamp biomicroscope, and an eyelid squeeze was performed to eliminate meibomian components that were heated by the IPL. The eyelid squeeze can lead to better meibomian gland condition and elevate the efficiency of IPL in addressing DED. Then, artificial tears, topical fluorometholone, and carbomer ointment were applied. Afterward, hyaluronic acid artificial tears (Systane® Hydration UD, Alcon, Fort Worth, Texas, United States), topical steroids (Fusone, Aseptic Innovative Medicine Co., Ltd., Taoyuan Dist., Taoyuan City, Taiwan), and overnight ointments (Duratears, Alcon, Fort Worth, Texas, United States) were used by all patients.

Dry eye evaluation

All of the individuals in this study received the same pre-IPL and post-IPL exams. Their history of ophthalmic diseases and ocular surgery (except KRS) was recorded in their medical documents. The pretreatment DED exams measured the uncorrected visual acuity (UDVA), cycloplegic refraction of sphere power, and cylinder power using an autorefractor (KR-8900, Topcon, Itabashi-ku, Tokyo, Japan), and the intraocular pressure (IOP) test was performed using a pneumatic tonometer (NT-530, NIDEK, Gamagori, Aichi, Japan). The cycloplegia refraction was presented as the spherical equivalent (SE) in this study, which was the sphere power plus half of the cylinder power. In regard to the DED-related factors, the non-invasive tear break-up time (NITBUT), the lipid thickness, the eyelid closure rate, the tear meniscus height (TMH), and the meibomian gland loss rate were obtained using one automatic ocular surface analyzer (SBM Device, SBM Sistemi, Strada Torino, Orbassano, Italy). In addition, the Schirmer I test without anesthesia and ocular surface staining using fluorescein were also performed in all individuals. The degree of ocular surface staining was based on the Oxford Scheme. In regard to the subjective DED symptoms, dryness, itchiness, grittiness, burning sensation, foreign body sensation, soreness, photophobia, discharge, and redness were listed in the medical documents. The post-treatment exams after IPL treatment measured the UDVA, IOP, and manifest refraction using identical devices. In addition, fluorescein staining, the Schirmer I test, NITBUT, and subjective DED symptoms were examined after the IPL treatment. The results of DED-related examinations performed before and six months after the IPL treatment were obtained in this study.

Statistical analysis

IBM SPSS Statistics software, version 20.0 (IBM Corp., Armonk, New York, USA), was used for statistical analysis in this study. The statistical power of this study was 0.66 with a 0.05 alpha value and a medium effect size generated using G∗power version 3.1.9.2 (Heinrich Heine Universität at Düsseldorf, Germany). The Shapiro-Wilk test was used to confirm the normality of the data, and the results presented the non-normal distributions of all data in this study (all P < 0.05). Descriptive analysis was used for the initial features of the KRS and non-KRS groups, and the Mann-Whitney U test and chi-square test were used to compare the pre-treatment factors and post-treatment outcomes between the two groups. The generalized linear model was used to compare the effect of IPL treatment between the two groups regarding the improvement of DED-related symptoms, Schirmer I test, NITBUT, and ocular surface staining. The adjusted odds ratio (aOR) and a 95% confidence interval (CI) were calculated after adjusting the age, sex, and SE in the analysis model. The multiple DED-related symptoms mean more than three DED-related symptoms. In the next step, we defined the poor outcome of IPL treatment as one of the following conditions: (1) no relief of DED-related symptoms three months after the IPL treatment, (2) a less than 3 mm increment of the Schirmer I test, (3) a less than three-second NITBUT increment, and (4) a less than 1 degree decrement of the fluorescein staining score. After that, the generalized linear model was used again to evaluate the potential risk factor of poor IPL treatment outcome in both the KRS group and the non-KRS group. In this study, the statistical significance was set as P < 0.05, and a P-value lower than 0.001 was presented as P < 0.001.

## Results

The baseline features of the KRS group and the non-KRS group are presented in Table 1. The mean age was 46.64±13.77 years old and 48.32±15.58 years old in the KRS and non-KRS groups, respectively, without a significant difference (P = 0.734). An older age would lead to a higher rate of DED development and progression. Also, the distributions of gender, ocular disease, and previous ophthalmic surgery between the two groups also showed a non-significant difference (all P > 0.05). Concerning the ophthalmic parameters, mean cycloplegia SE was significantly lower in the KRS group than in the non-KRS group (-1.24±0.88 versus -2.62±1.45, P = 0.004). The other ophthalmic parameters before the IPL treatment did not show significant differences between the two groups (all P > 0.05) (Table 1).

**Table 1:** The initial characteristics of the two groups D: diopter; DED: dry eye disease; KRS: keratorefractive surgery; N: number; SD: standard deviation; SE: spherical equivalent; NITBUT: non-invasive tear break-up time; TMH: tear meniscus height; UDVA: uncorrected distance visual acuity; * denotes significant difference between the two groups according to chi-square test (for sex, ocular disease, ocular surgery, lipid thickness, ocular surface staining, and DED-related syndrome) and Mann–Whitney U test (for others).

Feature	KRS group (N: 11)	Non-KRS group (N: 35)	P
Age (years, mean ± SD)	46.64±13.77	48.32±15.58	0.734
Sex (male: female)	4:7	14:21	-
Ocular disease	-	-	0.281
Retinal disease	1	0	-
Glaucoma	0	1	-
Other	0	1	-
Ocular surgery	-	-	0.171
Retinal surgery	1	0	-
Glaucoma surgery	0	1	-
UDVA (LogMAR)	0.16±0.20	0.34±0.29	0.068
Cycloplegia SE (D)	-1.24±0.88	-2.62±1.45	0.004*
IOP	15.27±4.31	15.86±4.17	0.672
NITBUT	6.54±3.85	7.13±3.64	0.658
Eyelid closure rate	92.33±8.42	91.97±9.03	0.891
Lipid thickness	-	-	0.734
Grade A-C	5	19	-
Grade D-E	6	16	-
TMH	0.28±0.15	0.30±0.16	0.726
Meibomian gland loss rate	32.62±18.72	31.68±17.35	0.845
Schirmer I test	9.60±2.49	9.93±2.32	0.673
Ocular surface staining	-	-	0.963
Grade 0-3	7	22	-
Grade 4-6	4	13	-
DED-related syndrome	-	-	0.504
1	2	13	-
2	5	12	-
≥ 3	4	10	-

Six months after the IPL treatment, the UDVA and IOP showed similar values between the two groups (both P > 0.05), and the cycloplegia SE was still lower in the KRS group than the non-KRS group (P = 0.002). About the improvement of DED, the post-treatment NITBUT was significantly higher in the non-KRS group than the KRS group (11.54±2.75 versus 7.68±3.62, P < 0.001) (Table 2). The other DED-related parameters did not present significant differences between the two groups (all P > 0.05) (Table 2). Regarding the trend of DED improvement, NITBUT (aOR: 2.517, 95% CI: 1.629-4.283, P < 0.001) and ocular surface staining (aOR: 1.841, 95% CI: 1.194-2.606, P = 0.001) presented better improvement in the non-KRS group (Table 3). However, the improvement of tear secretion and DED-related symptoms did not reveal significant differences between the groups (both P > 0.05) (Table 3).

**Table 2:** Ophthalmic parameters of the two groups after the intense pulsed light treatment DED: dry eye disease; IOP: intraocular pressure; KRS: keratorefractive surgery; N: number; SE: spherical equivalent; UDVA: uncorrected distance visual acuity; * denotes significant difference between the groups according to chi-square test (for ocular surface staining and DED-related syndrome) and Mann–Whitney U test (for others).

Outcome	KRS group (N: 11)	Non-KRS group (N: 35)	P
UDVA	0.15±0.18	0.32±0.29	0.073
SE	-1.25±0.86	-2.66±1.41	0.002*
IOP	16.01±4.58	15.34±4.09	0.622
Schirmer I test	11.38±2.67	12.85±2.29	0.081
NITBUT	7.68±3.62	11.54±2.75	<0.001*
Ocular surface staining	-	-	0.284
Grade 0-3	8	28	-
Grade 4-6	3	7	-
DED-related syndrome	-	-	0.512
1	3	16	-
2	4	11	-
≥ 3	4	8	-

**Table 3:** The trend of dry eye improvement between keratirefractive surgery and non-keratorefractive surgery populations aOR: adjusted odds ratio; CI: confidence interval; DED: dry eye disease; KRS: keratorefractive surgery; NITBUT: non-invasive tear break-up time; * denotes significant improvement in the non-KRS group compared to the KRS group according to the generalized linear model.

Outcome (reference: KRS group)	aOR	95% CI		P
		Lower	Upper	
Schirmer I test	1.332	0.938	1.523	0.143
NITBUT	2.517	1.629	4.283	<0.001*
Ocular surface staining	1.841	1.194	2.606	0.001*
DED-related syndromes	1.173	0.847	1.452	0.397

As for the pre-treatment risk factors for poor response after IPL treatment, low NITBUT, low TMH, high ocular surface staining, and multiple DED-related symptoms were correlated to poor treatment outcome in the KRS population (all P < 0.05) (Table 4). On the other hand, low NITBUT and high ocular surface staining were associated with poor treatment outcome in the non-KRS population (both P < 0.05) (Table 5).

**Table 4:** The correlation between pre-treatment parameters to poor outcome after intense pulse light treatment in eye with previous keratorefractive surgery aOR: adjusted odds ratio; CI: confidence interval; DED: dry eye disease; NITBUT: non-invasive tear break-up time; TMH: tear meniscus height; * denotes significant correlation to poor treatment outcome according to the generalized linear model.

Factor	aOR	95% CI		P
		Lower	Upper	
Low NITBUT	3.335	2.018	6.247	<0.001*
Low eyelid closure rate	1.285	0.672	1.941	0.532
Low lipid thickness	1.398	0.724	1.671	0.566
Low TMH	1.627	1.113	2.495	0.012*
High meibomian gland loss rate	1.369	0.992	1.788	0.057
Low Schirmer I test	1.299	0.926	1.573	0.071
High ocular surface staining	2.338	1.219	3.524	<0.001*
Multiple DED symptoms	1.527	1.063	1.949	0.004*

**Table 5:** The correlation between pre-treatment parameters to poor outcome after intense pulse light treatment in eye without previous keratorefractive surgery aOR: adjusted odds ratio; CI: confidence interval; DED: dry eye disease; NITBUT: non-invasive tear break-up time; TMH: tear meniscus height; * denotes significant correlation to poor treatment outcome according to the generalized linear model.

Factor	aOR	95% CI		P
		Lower	Upper	
Low NITBUT	2.154	1.477	2.982	<0.001*
Low eyelid blink rate	1.242	0.816	1.694	0.265
Low lipid thickness	1.135	0.767	1.583	0.321
Low TMH	0.988	0.795	1.446	0.228
High meibomian gland loss rate	1.345	0.944	1.760	0.121
Low Schirmer I test	1.143	0.861	1.499	0.155
High ocular surface staining	1.627	1.108	2.274	0.003*
Multiple DED symptoms	1.085	0.836	1.269	0.335

## Discussion

Briefly, patients with general DED showed a better DED improvement compared to DED patients who previously underwent KRS in regard to NITBUT and ocular surface staining. Moreover, low pre-treatment NITBUT and high pre-treatment ocular surface staining scores were correlated with poor outcomes after IPL treatment in both populations. In addition, low TMH and multiple DED-related symptoms were associated with worse IPL treatment outcomes in the KRS population.

Certain mechanisms can result in the formation of DED, according to previous publications [8, 18]. Inflammatory biomarkers can trigger DED, and interleukin levels are enhanced in people with DED [19]. In addition, both tumor necrosis factor and interferon families had higher concentrations in the case of DED [20]. In addition, reduction in ocular surface homeostasis and diminished tear film stability could fundamentally increase the risk of DED [1]. Reduced tear film stability contributes to corneal epithelium insult, triggers inflammatory cytokine release, and causes tear film osmolarity to increase, which ultimately results in an unstable tear film, the so-called vicious cycle of DED development [21]. In addition to the above mechanisms, oxidative stress is an important component of DED development [22]. According to previous experience, high oxidative stress would destroy the structure of DNA and result in the lipid peroxidation process and the subsequent development of DED [23]. On the other hand, goblet cell loss due to chemical burns and autoimmune disorders could diminish mucin secretion and result in mucin-deficient DED [18]. Corrupted corneal epithelium is accompanied by poor tear film stability and frequent DED-related symptoms. The two disorders are frequently present in patients with DED [5, 8]. KRS could cause limbal cell damage, elevated inflammatory cytokine levels, and corneal nerve damage, and all of the above components would contribute to a high degree of tear evaporation, inadequate tear film stability, and subsequent DED [24]. Although the duration of post-KRS DED may not be long, there were some cases that experienced prolonged DED after KRS [24]. In addition, DED after KRS had a higher risk of superficial keratitis and ocular discomfort in previous studies [25, 26]. Accordingly, we speculate that the presence of KRS may contribute to the lower effectiveness of DED management, including the IPL treatment. This hypothesis was supported by the results of this study.

DED patients who previously underwent KRS had worse NITBUT and ocular surface damage recovery after IPL treatment compared to patients with general DED, and the worse NITBUT and ocular surface damage would contribute to low optical quality and high frequency of ocular irritation, which may reduce long-term quality of life and visual acuity of these patients. In a previous study, IPL treatment resulted in a significant improvement in both DED signs and symptoms in DED patients who underwent KRS [27]. In addition, the combination of IPL treatment, sodium hyaluronate, and a heated eye mask can effectively reduce the severity of post-KRS DED, according to an earlier publication [16]. Still, studies evaluating the overall effectiveness of IPL treatment in cases of post-KRS DED and cases of general DED were rare. To our knowledge, this may be the first study to demonstrate the relatively worse therapeutic outcome of IPL treatment in the case of post-KRS DED than in the case of general DED. In addition, the pre-treatment ophthalmic conditions between the two groups were grossly similar, except the KRS group exhibited a lower degree of cycloplegia SE; thus, the homogeneity of ocular status between the two groups may be acceptable. Moreover, we adjusted some predisposing factors for DED development, including age and sex, in the multivariable analysis to reduce their effect on DED treatment and recovery. As a consequence, the overall therapeutic effectiveness of IPL treatment may indeed be worse in post-KRS DED, considering the DED symptoms. The IPL treatment had a thermal effect on the ocular surface [12], and the secretion of lipid from the meibomian gland could improve and increase tear film stability, which is a crucial factor for DED development and progression [28]. However, in the patients who underwent KRS, tear film stability and the condition of the ocular surface would be negatively affected [29]. As a consequence, the related parameters, including NITBUT and ocular surface staining in the KRS population, may not improve as quickly as those in the general population.

Concerning pre-treatment risk factors for the poor outcome of IPL treatment, low pre-treatment NITBUT, low pre-treatment TMH, a high pre-treatment ocular surface staining score, and multiple pre-treatment DED symptoms were correlated with worse treatment outcomes in the KRS group of this study. This correlation was rarely reported in the literature. In previous publications, lower tear breakup time is a critical factor in the development of DED [20]. Accordingly, both conditions may affect recovery after IPL treatment. On the other hand, a reduced tear volume may be associated with a lower improvement of DED symptoms in patients who previously underwent KRS, since the KRS would reduce tear secretion integrity [29]. Except for DED-related signs, multiple DED-related symptoms, which means more than three DED-related symptoms, were also correlated with the poor outcome of IPL treatment. It is possible that patients who underwent KRS and presented with multiple DED-related symptoms had elevated corneal nerve sensitivity; thus, the symptom reduction after the IPL treatment was lower compared to the non-KRS population, and residual DED-related symptoms constituted the poor-response criteria in this study. On the other hand, only the low pre-treatment NITBUT and high pre-treatment ocular surface staining score were associated with poor response after IPL treatment in the non-KRS group. Because the two parameters are crucial components in advanced DED status [6], it is reasonable that a higher degree of these two parameters relates to poor IPL results in the population with general DED. Still, the presence of fewer risk factors for poor outcome in the non-KRS group compared to the KRS group may further indicate the challenging course of treatment in the KRS population.

Regarding the effectiveness of IPL treatment in this study, tear secretion function improved by about 30% after three IPL treatments, and the improvement in the NITBUT was statistically significant compared to the pre-treatment NITBUT. In a previous study, there was a non-significant improvement in tear secretion function after IPL treatment [30]. The tear secretion function of this study may be comparable to previous publications. In addition, the NITBUT showed a significant improvement after the IPL treatment in this study, where NITBUT increases of approximately 50% and 20% were observed in the non-KRS and KRS groups after the IPL treatment, respectively. In an earlier study, NITBUT status also improved after IPL treatment [30], and this study showed similar findings to the previous study. The corneal staining score in this study also improved, which is similar to the results in previous studies [13, 30]. On the contrary, DED-related symptoms did not subside significantly after the IPL treatment, and many patients still had multiple DED symptoms. However, DED-related symptoms improved significantly after IPL treatment in an earlier article [13]. Generally, the effectiveness of the IPL treatment in this study may not be inferior to previous publications, although a lower symptom reduction was observed.

There were some limitations in this study. Firstly, the case numbers of this study were too low, with only 46 eyes used in the analysis. The lack of adequate case numbers may cause statistical bias and severely reduce the accuracy and integrity of our results. In addition, the retrospective nature of this study could decrease the homogeneity of the study population, although all the preoperative parameters between the KRS and non-KRS groups were statistically identical, except the cycloplegia SE. Moreover, several items of the automatic ocular surface analyzer, including post-treatment TMH and post-treatment meibomian gland loss rate, were not examined due to the retrospective design; thus, a longitudinal analysis for these parameters could not be achieved. Finally, nearly all the individuals in this study are Han Taiwanese, and the external validity of this study could be reduced.

## Conclusions

In conclusion, the DED individuals with previous KRS showed worse NITBUT and ocular surface staining improvements compared to the general DED. Furthermore, the DED population with previous KRS is associated with more risk factors for poor outcomes after IPL treatment than the general DED population. Consequently, additional management may be applied to DED individuals with previous KRS who are scheduled for IPL treatment for a better therapeutic effect. Further large-scale prospective research with an adequate follow-up period to survey the suitable combined treatment for DED patients with previous KRS is mandatory.
